# Extent of the protection afforded by histo-blood group polymorphism against rotavirus gastroenteritis in metropolitan France and French Guiana

**DOI:** 10.3389/fmicb.2023.1141652

**Published:** 2023-03-10

**Authors:** Lydie Masson, Laure Barbé, Fanny Henaff, Tasnuva Ahmed, Béatrice Le Moullac-Vaidye, Cécile Peltier, Sarah S Marchand, Pauline Scherdel, Marie-Anne Vibet, Nathalie Ruvoën-Clouet, Narcisse Elenga, Berthe-Marie Imbert-Marcille, Christèle Gras-Le Guen, Jacques Le Pendu

**Affiliations:** ^1^Department of Pediatrics, University Hospital of Nantes, Nantes, France; ^2^Nantes Université, Inserm, CNRS, Immunology and New Concepts in Immunotherapy, INCIT, Nantes, France; ^3^Department of Pediatrics, Centre Hospitalier Andrée Rosemon, Cayenne, France; ^4^International Centre for Diarrheal Disease Research, Dhaka, Bangladesh; ^5^Center for Research in Transplantation and Translational Immunology, Inserm, Nantes Université, Nantes, France; ^6^Virology Department, University Hospital of Nantes, Nantes, France; ^7^Clinical Investigation Center (CIC004), Inserm, University Hospital of Nantes, Nantes, France; ^8^Clinical Research Department, University Hospital of Nantes, Nantes, France; ^9^Ecole Nationale Vétérinaire, Agroalimentaire et de l'Alimentation, Oniris, Nantes, France

**Keywords:** rotavirus, gastroenteritis, genetic susceptibility, histo-blood group antigens, disease severity, metropolitan France, French Guiana

## Abstract

Human rotaviruses attach to histo-blood group antigens glycans and null alleles of the *ABO*, *FUT2* and *FUT3* genes seem to confer diminished risk of gastroenteritis. Yet, the true extent of this protection remains poorly quantified. Here, we conducted a prospective study to evaluate the risk of consulting at the hospital in non-vaccinated pediatric patients according to the ABO, FUT2 (secretor) and FUT3 (Lewis) polymorphisms, in Metropolitan France and French Guiana. At both locations, P genotypes were largely dominated by P [8]-3, with P [6] cases exclusively found in French Guiana. The FUT2 null (nonsecretor) and FUT3 null (Lewis negative) phenotypes conferred near full protection against severe gastroenteritis due to P [8]-3 strains (OR 0.03, 95% CI [0.00–0.21] and 0.1, 95% CI [0.01–0.43], respectively in Metropolitan France; OR 0.08, 95% CI [0.01–0.52] and 0.14, 95%CI [0.01–0.99], respectively in French Guiana). Blood group O also appeared protective in Metropolitan France (OR 0.38, 95% CI [0.23–0.62]), but not in French Guiana. The discrepancy between the two locations was explained by a recruitment at the hospital of less severe cases in French Guiana than in Metropolitan France. Considering the frequencies of the null ABO, Secretor and Lewis phenotypes, the data indicate that in a Western European population, 34% (95% CI [29%; 39%]) of infants are genetically protected against rotavirus gastroenteritis of sufficient severity to lead to hospital visit.

## Introduction

Group A rotaviruses (RVs) are the most common cause of acute gastroenteritis (AGE) in children under 5 years. Prior to the availability of vaccines, they caused the death of more than 400.000 children every year. Currently, mortality figures have dropped below 150.000 deaths/year (Tate et al., 2016; Troeger et al., 2018). However, RVs still represent a major public health problem in high-income countries where vaccination has not been implemented into the national vaccination program (Ardura-Garcia et al., 2021). Thus in France, rotavirus infection is responsible for an estimated 300.000 acute diarrhea episodes among young children below 5 years, about 130.000 visits to general practitioners and an annual incidence of rotavirus estimated to result in over 3% of gastroenteritis hospitalizations, representing roughly 12.000 children (Melliez et al., 2005; Parez et al., 2013; Lamrani et al., 2017).

Rotaviruses (RVs) are double-stranded segmented and non-enveloped RNA viruses that belong to the *Reoviridae* family. At least ten different species of RVs (A–J) are defined, the vast majority of human cases being caused by group A rotaviruses (RVAs; Matthijnssens et al., 2011; Crawford et al., 2017). The two outer capsid proteins VP7 and VP4 are commonly used for further classification of strains, defining the G and P genotypes, respectively (Matthijnssens et al., 2011). Currently, 42 G and 58 P genotypes have been identified in human and animals, among which six G genotypes (G1, G2, G3, G4, G9, and G12) and 3 P genotypes (P [4], P [6], P [8]) are globally prevalent (Desselberger, 2014; Crawford et al., 2017; Park et al., 2019). Among the P genotypes, P [8] is the most prevalent. Distinct lineages P [8]-1, -2, -3, -4 are recognized and the current dominant P [8]-3 has been subdivided into six sub-lineages P [8]-3.1–P [8]-3.6 (da Silva et al., 2013).

Several scores have been proposed to assess the severity of rotavirus AGE. The Vesikari score (Ruuska and Vesikari, 1990) and Clinical Dehydration Scale (CDS; Friedman et al., 2004) are the most commonly used. They allowed observation of a large variability in disease severity, severe forms representing between 30 and 80% of RV gastroenteritis. A recent meta-analysis of published data from high-income countries indicated that the mean proportion of emergency department visits and hospitalizations for acute RV gastroenteritis represented about half of the total diarrheal cases but results were highly variable both between and within countries (Ardura-Garcia et al., 2021).

After exposure to RVA, individuals display differing susceptibility with only half becoming symptomatic and developing AGE (Desselberger, 2014; Crawford et al., 2017). This variability has also been found in vaccine responses since the live attenuated vaccines are more effective in high-income countries than in low-income countries (Burnett et al., 2020). Part of this variability may be explained by histo-blood group antigens (HBGAs), which are common genetic polymorphisms. In the human small intestine, HBGAs are synthesized by enzymes encoded by the *FUT2*, *FUT3* and *ABO* genes and genetic polymorphisms at each of these loci are responsible for the Secretor, Lewis and ABO phenotypes. Thus, null alleles of *FUT2* generate the nonsecretor phenotype, whilst null alleles of *FUT3* locus generate the Lewis negative phenotype. Null alleles of the *ABO* gene are responsible for blood group O, while co-dominant A and B alleles are responsible for the A, B and AB blood groups (see HBGAs biosynthesis; Supplementary Figure S1). The relative frequencies of wild type and null alleles at each of these loci are variable across geographical areas and human subpopulations (Marionneau et al., 2001; Yamamoto et al., 2012; Stowell and Stowell, 2019). Landmark studies showed that VP8*, the outermost domain of the VP4 spike protein of human RVA strains, could specifically bind to HBGAs and several studies showed that nonsecretor children (FUT2 null) presented a much lower risk of disease due to the common P [8] strains than secretor children who express the cognate VP8* ligand (Imbert-Marcille et al., 2014; Nordgren et al., 2014; Van Trang et al., 2014; Kambhampati et al., 2016; Zhang et al., 2016; Yang et al., 2017; Farahmand et al., 2021; Wang et al., 2021). Likewise, an effect of the Secretor phenotypes on vaccine take has been observed for both the live-attenuated RotaTeq and Rotarix vaccines, with nonsecretor children presenting diminished responses in comparison with secretor children (Kazi et al., 2017; Bucardo et al., 2018; Lee et al., 2018; Armah et al., 2019; Magwira et al., 2020). Yet, contradictory reports from Tunisia, Bangladesh and the Amazonia region failed to uncover any significant effect of the Secretor phenotypes on RVA infection (Ayouni et al., 2015; Lee et al., 2018; Olivares et al., 2021). Fewer studies have been conducted to evaluate the effect on infection of the Lewis phenotypes. A study by Nordgren et al. (2014) conducted in Burkina-Faso revealed that infection by P [6] strains were less frequent among Lewis positive children in comparison with Lewis negative children. This underscores the importance of strain typing since P [6] strains binding to glycans is hindered by the presence of the Lewis fucose (Huang et al., 2012; Barbé et al., 2018). More recently, additional studies evaluating the risk of P [8] gastroenteritis found a lower risk for Lewis negative individuals, in accordance with the recognition of the Lewis fucose by these strains (Yang et al., 2017; Pérez-Ortín et al., 2019; Farahmand et al., 2021). Yet, no significant effect was observed in the Amazonia study (Olivares et al., 2021). Few studies evaluated the impact of ABO phenotypes on RVA infection and AGE. Nonetheless, a lower risk of disease for blood group O children was reported in Spain and a higher risk for blood group A children in comparison with those of blood group O was observed in Egypt (Elnady et al., 2017; Pérez-Ortín et al., 2019). In contrast, no effect of the ABO phenotype was evident in studies from Taiwan or from the Amazonia region (Yang et al., 2017; Olivares et al., 2021).

The aim of the present study was to clarify these seemingly contradictory data and to evaluate the real extent of the protection afforded by HBGA polymorphisms on dominant P [8] strains. To this aim, we performed a prospective study at two sites, one in Nantes, Metropolitan France, Western Europe, the other one in Cayenne, French Guiana, South America. Parallel study of the two sites allows for a comparison of populations with distinct genetic backgrounds living in different environments where circulating strains may differ.

## Population and methods

### Study design, participants and collection of samples

A case–control and multicenter study, based on a prospective genetic epidemiology study was conducted in parallel in Nantes and Cayenne. The study included children from birth to 16 years old, having consulted at the pediatric Emergency Department (ED) of Nantes University Hospital, between March 2017 and June 2019 and at the Cayenne Hospital, between August 2016 and September 2020. We studied the impact of genetic polymorphisms on (i) the RV gastroenteritis occurrence by comparing a group of children seen for RV gastroenteritis (cases) and children hospitalized for reasons other than gastroenteritis, including newborns from the maternity ward (controls), and (ii) the severity of RV gastroenteritis among cases. Since extant populations at both locations are an admixture of people from geographically distinct regions of origins, control children were matched by site and ancestry to avoid potential biases in HBGA polymorphism across populations. Parent informed consents were obtained after giving them written information.

Inclusion criteria for cases were children aged from birth to 5 years old presenting to the emergency department for RV gastroenteritis (cases), commonly defined by at least three soft or liquid stools with or without episodes of vomiting in 24 h, or children from birth to 16 years old for controls. Cases were confirmed by the identification of RV in stool sample. Exclusion criteria were presence of a co-infection (viral or bacterial) for cases only or history of RV vaccination and unavailable samples for genetic or virology testing for both cases and controls. Enteric bacterial co-infection was excluded through a classical negative coproculture as well as negativity for the major enteric bacteria and clinical signs. Controls were diarrhea-free and not presenting for the treatment of infectious causes enrolled in parallel with cases. The study was filed as the GASTROVIM project [reference RC15-0329, ClinicalTrials.gov identifier NCT02902445]. It was approved by the French ethic committee of OUEST V (December 14, 2015, no. 2016-A01047-44). Date for a report: June 30, 2022.

### Data collection

Pediatricians completed a standardized questionnaire after medical examinations. This questionnaire included information about demographic data (age, sex, and parents’ ancestry), vaccine status, symptoms duration and frequency, clinical signs included in the clinical dehydration scale proposed by Friedman and collaborators such as general appearance, eyes, mucous membranes and tears, anterior consultations and treatments (Friedman et al., 2004).

### Gastroenteritis severity scores

The children were classified in different clinical severity forms of RV gastroenteritis. In ED, children are usually considered with a severe clinical form in case of clinical signs of dehydration, profuse digestive symptoms, biological abnormalities (such as renal failure, clear serum electrolyte abnormalities), or hemodynamic disorders. These items correspond to usual clinical hospitalization criteria. Because of the poor diagnostic value of the usual scores (lack of specificity of the Vesikari score and lack of sensitivity of the CDS score), we generated a composite clinical score, combining items of those two gastroenteritis severity scores. Our score was based on the CDS, the percentage of dehydration (according to the WHO criteria), and the maximal number per day of liquid stools and of vomiting. The patients were thus classified into severe or mild to moderate forms as following: a patient was classified into severe forms if he/she had a CDS equal or above 4, a percentage of dehydration equal or above 7%, a maximal number of liquid stool per day equal or above 8, or maximal number of vomiting per day equal or above 10. A more detailed description of the composite score will be given in an independent manuscript.

### Virus detection and P type genotyping

Nucleic acids were extracted from approximately 100 mg stool samples. RV was diagnosed by RT-PCR using the FTD Viral gastroenteritis kit (Fast Track Diagnostics, Esch-sur-Alzette, Luxembourg) to detect enteric viruses. For all samples that were RV positive, an 850-bp fragment of VP4 sequences was obtained from consensus primers and used for classification of circulating strains at the P-type level. When cycle threshold (Ct) values were < 18–20, a 1-step RT-PCR was performed using the One-Step PrimeScript RT-PCR kit (Takara) with 5 μl nucleic acids extract and ROTA CON3 M13F/ROTA CON2 M13R primers. When Ct values were > 18–20, a first RT-PCR was performed using ROTA CON3F/ROTA CON2R and 5–10 μl nucleic acid extract, followed by a nested PCR with ROTA VP4 M13F/ROTA VP4 M13R primers and Premix Ex Taq kit (Takara). In cases where no amplification was detected, a first RT-PCR was performed with ROTA VP4F/ROTA VP4R primers followed by the nested PCR as above. Primer sequences are given in the Supplementary material. Purified amplicons were then sequenced using the Big Dye version 3.1 Cycle Sequencing Kit (Applied Biosystems) on an ABI 3730 XL Sequence Analyser and consensus sequences in FASTA format were obtained using the SeqScape^™^ Sofware v4.1 (Thermofisher). Genotypes were ascribed from a phylogenetic analysis performed using the MEGA X software with references sequences of the known genotypes (see Supplementary material).

### ABO, Secretor and Lewis phenotyping and genotyping

#### Histo-blood group antigens phenotyping

Histo-blood group antigens (HBGAs), phenotypes were determined from buccal swabs specimens by enzyme-linked immunosorbent assay (ELISA) as described previously (Marionneau et al., 2005). Buccal swabs immersed in PBS were first boiled for 10 min and then used at a dilution of 1:50 in a 0.1 M carbonate/bicarbonate buffer (pH 9.6) to coat 96-well microtiter plates (Maxisorp Nunc-Immuno plates, Thermo Scientific, CA, United States). Primary anti-carbohydrate monoclonal antibodies anti-A (ABO1 9113D10, Diagast, Loos, France), anti-B (B49), anti-Le^a^ (7LE) and anti-Le^b^ (2-25LE; Thermo Scientific, CA, United States) diluted at 1:400 in 5% milk/PBS were incubated for 1 h at 37°C. The lectin biotin-conjugated UEA-1 (Ulex Europaeus Agglutinin I—Vector Laboratories, CA, United States) was additionally used to detect the H antigen. Peroxidase–conjugated secondary reagents were used (Vector Laboratories, CA, United States) and reactions were developed with a 3,3′,5,5′-Tetramethylbenzidine kit (BD OptEIA, BD Biosciences). Previously well-defined A, B, O, secretor and Lewis types saliva samples were included in each plate as controls. The cutoff value was defined as a twofold increase in absorbance value compared to the mean of two negative control samples (wells with PBS only).

#### ABO, FUT2 and FUT3 genotyping

Because ABO, Secretor and Lewis phenotyping from buccal swabs may be affected by unknown factors, genotyping was used for confirmation. In presence of a mismatch between the phenotype and genotype, the case had to be discarded from the analysis. Single nucleotide polymorphisms (SNPs) in the FUT2 and FUT3 genes were essentially investigated as described previously (Marionneau et al., 2005; Loureiro Tonini et al., 2020) with a modification for the Lewis (FUT3) gene mutations. Here, two gene fragments encompassing the worldwide common mutations C314T, G508A, and T1067A were amplified by real-time PCR and submitted to Sanger DNA sequencing. The remaining common mutations T59G, T202G, were searched by PCR using sequence-specific primers, as previously described (Grahn et al., 2001). ABO genotyping was performed by real-time PCR using allele specific primers, as described by Muro et al. (2012).

### Statistical analysis

Description of the P-types distribution in Nantes and Cayenne was performed on 210 and 42 cases, respectively for whom P-type data was available. The demographic characteristics in cases and controls, the virology testing and the gastroenteritis symptoms in cases were compared by using Mann–Whitney and chi-square tests (or Fisher’s exact test), respectively. The impact of HBGA phenotypes on the risk of gastroenteritis was analyzed by using crude and adjusted logistic regression models in order to calculate odds ratios (OR) as well as their 95% confidence intervals (CIs). The Wald’s test was used for the adjusted logistic regression analysis. A multinomial logistic regression to study the relationship between disease severity and ABO phenotype was performed. The analysis was stratified by hospital. The fraction of children genetically protected from gastroenteritis of sufficient severity to require a consultation at the hospital in a Western European population was estimated using the data from Nantes. A value of *p* < 0.05 was considered statistically significant. To this aim the prevalence of Lewis, Secretor and ABO phenotypes observed in the control group was used. The population of children was partitioned into distinct groups according to the ABO, Lewis and Secretor phenotypes (see Supplementary Figure S2). In each group, the fraction of protected children was estimated from the probability of belonging to each group by using the prevalences observed in the control group and the deficit of cases compared to controls. The confidence interval was estimated assuming the number of cases in each group follows a binomial distribution. All analyses involved the use of R version 4.1.1. (R Foundation for Statistical Computing, Vienna, Austria). A value of *p* < 0.05 was considered statistically significant.

## Results

### Description of participants and of circulating strains

In Nantes, 388 children were enrolled onto the study, 228 with RV gastroenteritis and 160 controls, with 200 cases and 158 controls included in the final analysis (Supplementary Figure S3A). In Cayenne, among the 324 eligible children, including 189 cases and 137 controls, 49 patients and 120 controls were included (Supplementary Figure S3B).

**Figure 1:**
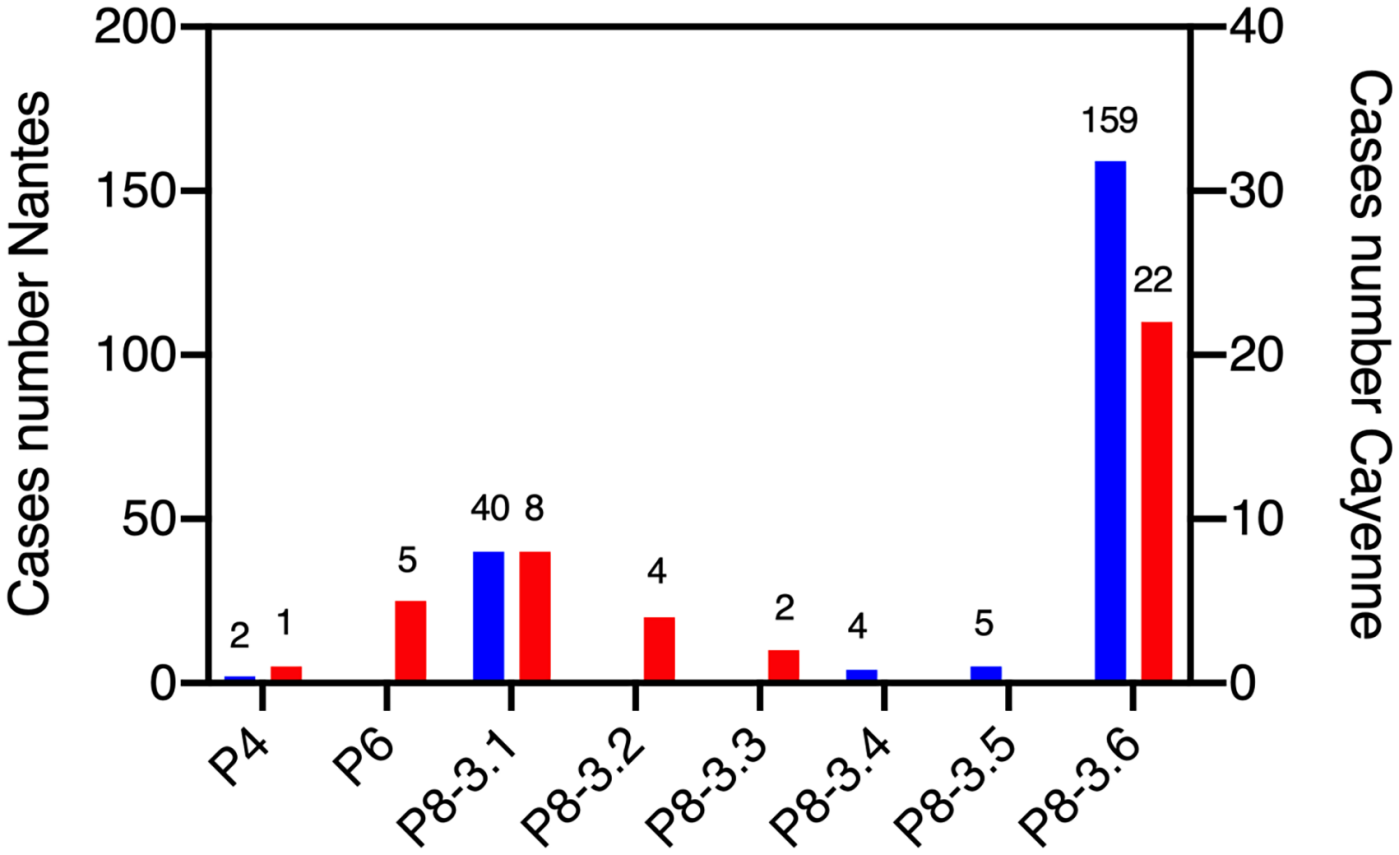
Distribution of rotavirus P genotypes at Nantes and Cayenne. Sequences of the VP4 protein region allowed for classification. Blue bars represent the number of cases in Nantes and red bars represent the number of cases in Cayenne. Number of sequences is shown above bars. All available P genotypes are shown, regardless of the availability histo-blood group antigen (HBGA) or clinical severity scores.

Comparison of VP8* sequences allowed sub-classification P[8]-3 strains into P[8]-3.1 to P[8]-3.6. Analyzing the distribution of P types in Nantes (*n* = 210) and Cayenne (*n* = 42) in all patients for whom sequences were available, including those without secretor, Lewis and blood group results, revealed that at both locations P[8]-3.6 and P[8]-3.1 dominated. Rarer P[8]-3.2 and P[8]-3.3 were only found in Cayenne, whilst P[8]-3.4 and P[8]-3.5 were exclusively detected in Nantes. At both sites P[4] strains were also rarely detected. P[6] strains were exclusively found in Cayenne, representing 12% of the total available sequences (Figure 1). Due to the low numbers of P[4] and P[6] strains, the HBGA association study was conducted on cases infected by P[8]-3 strains, regardless of the subtype.

### Demographics of participants

Mean ages of cases were 17.6 months and 21.3 months in Nantes and Cayenne, respectively. At both locations ages of controls (8.0 months and 8.3 months, respectively) were lower due to recruitment from the maternity wards. The majority of cases were over 6 m/o (88.0% in Nantes and 79.6% in Cayenne). In Nantes, the sex ratio was significantly different between cases and controls, females being slightly under-represented among cases. The same trend was visible in Cayenne, but did not reach significance. In Nantes, parents of European descent were the most represented. In Cayenne, the major ancestries were represented by various Amerindian groups, Haitians, Brazilians, and Creoles. No significant differences in ancestry were noted between cases and controls at each site (Table 1).

**Table 1:** Description of cases and controls in Nantes and Cayenne.

	Nantes				Cayenne			
	Total (*n* = 358)	Cases (*n* = 200)	Controls (*n* = 158)	Value of p^*^	Total (*n* = 169)	Cases (*n* = 49)	Controls (*n* = 120)	Value of p^*^
Age (months), mean ± SD	13.4 ± 12.9	17.6 ± 12.1	8.0 ± 11.7	**< 0.01**	12.1 ± 24.8	21.3 ± 20.8	8.3 ± 25.3	**< 0.01**
Age > 6 months, *n* (%)	240 (67.0)	176 (88.0)	64 (40.5)	**< 0.01**	59 (35.5)	39 (79.6)	20 (17.1)	**< 0.01**
Sex (Female), *n* (%)	135 (37.7)	64 (32.0)	71 (44.9)	0.02	57 (33.7)	13 (26.5)	44 (37.6)	0.23
Mother’s ancestries,*n* (%)				0.90				0.57
Europe	283 (79.0)	157 (78.5)	126 (80.6)		9 (5.6)	3 (6.2)	6 (5.3)	
Sub-Saharan Africa	24 (6.7)	12 (6.0)	12 (7.6)		1 (0.6)	0 (0.0)	1 (0.9)	
Middle East Africa	40 (11.2)	23 (11.5)	17 (10.8)		0 (0.0)	0 (0.0)	0 (0.0)	
Others	11 (3.0)	8 (4.0)	3 (1.9)		151 (93.8)	45 (93.8)	106 (93.8)	
Father’s ancestries, *n* (%)				0.91				0.68
Europe	276 (77.1)	151 (75.5)	125 (79.1)		5 (4.1)	1 (3.4)	4 (4.3)	
Sub-Saharan Africa	31 (8.7)	19 (9.5)	18 (7.6)		1 (0.1)	0 (0.0)	1 (1.0)	
Middle East Africa	41 (11.5)	23 (11.5)	12 (11.4)		0 (0.0)	0 (0.0)	0 (0.0)	
Others	10 (2.8)	7 (3.5)	3 (1.9)		117 (95.1)	28 (96.6)	89 (94.7)	

Significant *p* values are indicated in bold.

* Fisher’s exact test or Chi-squared test for qualitative data and Mann–Whitney test for quantitative data. Other ancestries at Cayenne were Amerindians, Haitians of African ancestry, Brazilians of African ancestry and Creoles.

### Gastroenteritis symptoms and clinical severity scores

Diarrhea and vomiting were the most common symptoms. Dehydration percentages, temperature, duration of diarrhea and vomiting as well as the need for rehydration, perfusion and maximal number of stools were similar in Nantes and Cayenne. Yet, vomiting maximal duration and hospitalization were significantly higher in Nantes than in Cayenne (Table 2). The majority of children presented with severe illness, but comparison of the two sites indicated a recruitment of less severe diarrheal patients in Cayenne than in Nantes (Table 3).

**Table 2:** Description of gastroenteritis symptoms.

	Nantes (*n* = 200)	Cayenne (*n* = 49)	Value of p^*^
Symptom duration (days), mean ± SD	3.2 ± 2.8	3.2 ± 2.7	0.93
Dehydration percentages, *n* (%)			0.19
< 5%	88 (46.8)	15 (37.5)	
5–10%	66 (35.1)	19 (47.5)	
> 10%	34 (18.1)	6 (15.0)	
Maximal temperature (°C), mean ± SD	38.5 ± 0.9	38.3 ± 1.1	0.23
Diarrhea, *n* (%)	195 (97.5)	48 (98.0)	1.00
Diarrhea duration (days), mean ± SD	2.7 ± 2.3	2.9 ± 2.3	0.22
Maximal stool number per day, mean ± SD	8.5 ± 7.0	6.0 ± 2.4	0.06
Vomiting, *n* (%)	192 (96.0)	43 (87.8)	**0.04**
Vomiting duration (days), mean ± SD	2.5 ± 1.6	2.9 ± 2.3	0.32
Maximal vomiting number per day, mean ± SD	8.7 ± 7.4	4.8 ± 3.8	**<0.01**
Rehydration, *n* (%)	138 (71.5)	29 (67.4)	0.58
Hospitalization, *n* (%)	173 (86.5)	30 (61.2)	**<0.01**
Perfusion, *n* (%)	148 (74.0)	28 (59.6)	0.07

Significant *p* values are indicated in bold.

* Fisher’s exact test or Chi-squared test for qualitative data and Kruskal–Wallis rank sum test for quantitative data.

**Table 3:** Patients in Cayenne were less severely affected than in Nantes.

	Nantes (*n* = 200)	Cayenne (*n* = 49)	Value of p^*^
Composite score, *n* (%)			**<0.01**
Benign spontaneous evolution	45 (22.5)	22 (41.4)	
High risk of dehydration or lack of appropriate care	155 (77.5)	27 (58.6)	

Significant *p* values are indicated in bold.

* Chi-squared test.

### Impact of HBGA polymorphisms on the risk of gastroenteritis and disease severity

In both Nantes and Cayenne, the secretor and Lewis phenotypes were strongly associated with non-susceptibility to the disease induced by P[8]-3 RV since the corresponding negative phenotypes were hardly represented among patients (Tables 4, 5). However, ABO phenotypes showed a different pattern. In Nantes, the frequency of blood group O was much lower among cases than among controls (20.5 vs. 40.5%). However, no such decrease of blood group O in the cases group could be observed in Cayenne. Since the P[8]-3 strains found in Nantes and Cayenne, as well as the distribution of ABO phenotypes were largely similar (Figure 1), that discrepancy between the two sites could only stem from either a socio-demographic effect or an environmental effect. As we observed a lower severity of AGE in Cayenne than in Nantes, we hypothesized that blood group O children may be susceptible to infection by P[8]-3 strains, but have a less severe disease outcome. The difference in blood group O frequency between the two sites steming from a selection bias in Nantes where more severely children would have been enrolled.

**Table 4:** Crude and adjusted logistic regression analysis shows high impact of histo-blood group antigen (HBGA) polymorphisms between cases and controls in Nantes.

	Cases (*n* = 200)	Controls (*n* = 158)	Crude odds ratio [95% CI] (ref = controls)	Value of p^*^	Adjusted odds ratio† [95% CI] (ref = controls)	Value of p^**^
Secretor phenotype (*n* = 358)
Secretor	199 (99.5%)	136 (86.1%)	ref		ref	
Non-secretor	1 (0.5%)	22 (13.9%)	0.03 (0.00–0.21)	**<0.001**	0.03 (0.00–0.26)	**0.001**
Lewis phenotype (*n* = 358)
Positive	198 (99.0%)	143 (90.5%)	ref		ref	
Negative	2 (1.0%)	15 (9.5%)	0.10 (0.01–0.43)	**<0.01**	0.12 (0.03–0.54)	**<0.01**
ABO phenotype (*n* = 358)
Non-O blood group	159 (79.5%)	94 (59.5%)	ref		ref	
O blood group	41 (20.5%)	64 (40.5%)	0.38 (0.23–0.62)	**<0.001**	0.39 (0.24–0.64)	**<0.001**

Significant *p* values are indicated in bold.

* Fisher’s exact test,

** Wald’s test,

† Adjusted on Secretor, Lewis and ABO phenotypes.

**Table 5:** Crude and adjusted logistic regression analysis shows impact of FUT2 and FUT3 polymorphisms between cases and controls in Cayenne.

	Cases (*n* = 49)	Controls (*n* = 120)	Crude odds ratio [95% CI] (ref = controls)	Value of p^*^	Adjusted odds ratio^‡^ [95% CI] (ref = controls)	Value of p^**^
Secretor phenotype (*n* = 169)
Secretor	48 (98.0%)	95 (79.2%)	ref		ref	
Non-secretor	1 (2.0%)	25 (20.8%)	0.08 (0.01–0.52)	**<0.01**	0.00 (0.00 – Inf)	0.99
Lewis phenotype^†^ (*n* = 168)
Positive	48 (98.0%)	104 (87.4%)	ref		ref	
Negative	1 (2.0%)	15 (12.6%)	0.14 (0.01–0.99)	**0.04**	0.14 (0.02–1.13)	0.07
ABO phenotype^†^ (*n* = 159)
Non-O blood group	25 (52.1%)	63 (55.8%)	ref		ref	
O blood group	23 (47.9%)	48 (44.2%)	1.21 (0.58–2.52)	0.61	1.00 (0.49–2.02)	0.99

Significant *p* values are indicated in bold.

* Fisher’s exact test,

** Wald’s test,

† Data missing for some individuals,

‡ Adjusted on Secretor, Lewis and ABO phenotypes (n = 158).

**Figure 2:**
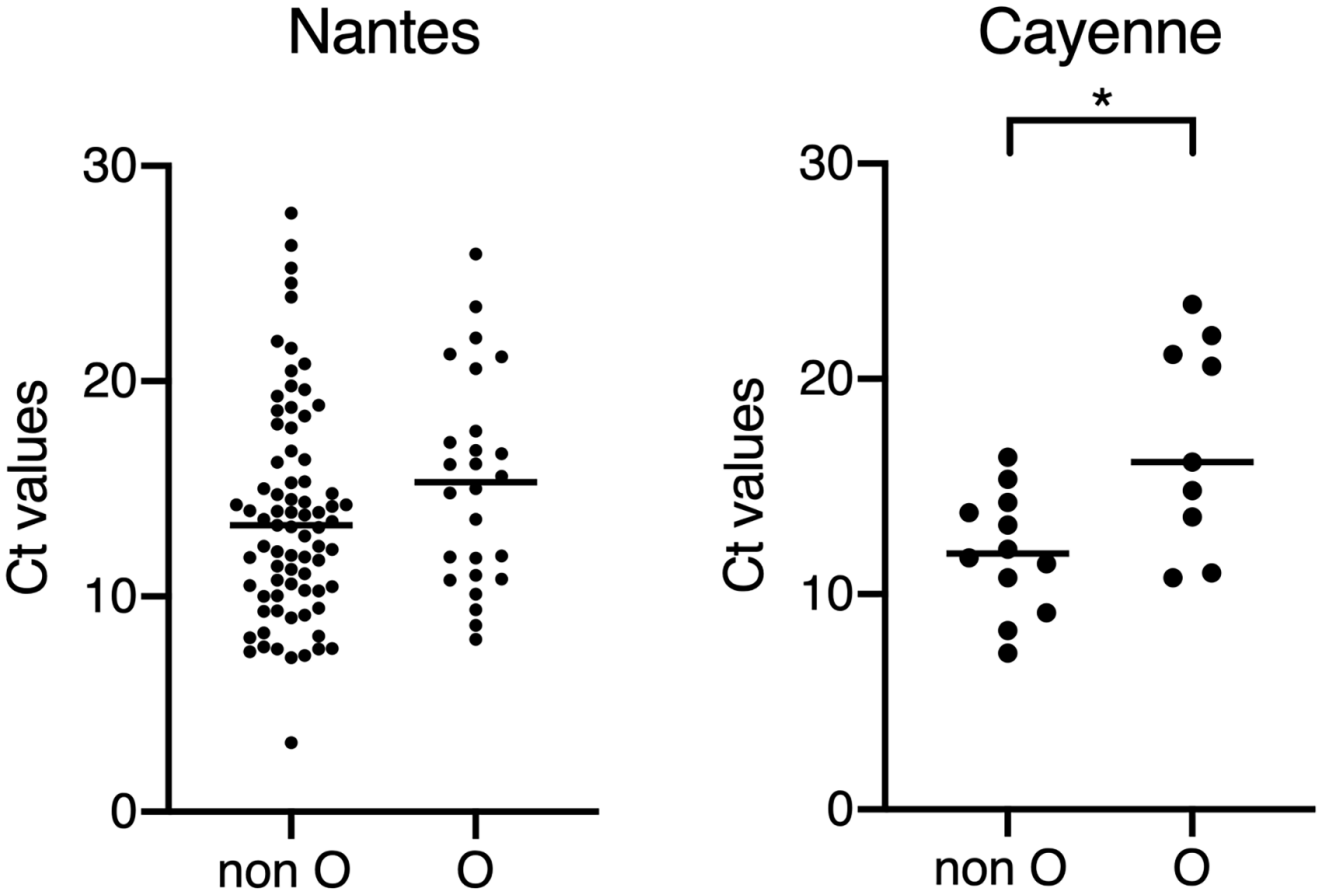
Comparison of Ct values between children of the O and non-O blood groups. Non-O blood groups correspond to A, B and AB blood groups. All samples for which Ct values could be obtained are shown, regardless of the availability HBGA or clinical severity scores. Higher Ct values correspond to lower amount of detected viral RNA, suggesting lower viral excretion and association with lower clinical severity of AGE (Bonacorsi et al., 2021). ^*^p < 0.05.

In order to test this hypothesis, we compared the Ct values of the PCR assay used for the virus detection between blood groups O and non-O cases both in Nantes and Cayenne (Figure 2) since low Ct values have recently been associated with symptomatic disease and severity (Bonacorsi et al., 2021). Regardless of blood groups, Ct values were not different between Nantes and Cayenne cases (mean values 14.2 vs. 14.6). In Cayenne but not in Nantes, Ct values of blood group O patients were significantly higher than those of non-O patients (17 vs. 12, *p* = 0.03), suggesting a relationship between blood group O and a lower excretion of virus. We next studied the relationship between disease severity and ABO phenotype in Nantes and Cayenne (Supplementary Tables S1 and S2). In Nantes, blood group O appeared significantly protective regardless of the degree of severity. Yet, in Cayenne, it was more frequent among non-severe cases in comparison with severe cases, although the difference did not reach significance due to the limited number of cases.

Finally, we estimated the fraction of children genetically protected from gastroenteritis of sufficient severity to require a consultation at the hospital in a Western European population. The frequency of children with HBGA phenotypes missing among cases was deduced from a comparison of those frequencies among controls (as shown on Supplementary Figure S2). The analysis revealed that 34% (95% CI [29%; 39%]) of children were naturally protected by HBGA polymorphisms. We did not perform the same analysis in Cayenne since the available number of cases was low and since the proportion of P[8]-3 RVA cases among total gastroenteritis cases was much lower than in Nantes due frequent infection or co-infection by other pathogens.

## Discussion

Despite the large diversity of RVs, only a few P types are responsible for the majority of cases worldwide. Accordingly, in the present study we found that P[8]-3 strains overwhelmingly dominated in both Nantes and Cayenne. Cases caused by P[4] were seldom encountered and P[6] strains were represented in Cayenne only. These observations are in accordance with previous data from Western Europe, including France and from South America, respectively (de Rougemont et al., 2016; Kaplon et al., 2018; Santos et al., 2018). Here, we confirm that both the Secretor and Lewis status are strongly associated with the risk of P[8]-3 AGE at both geographical locations, consistent with the carbohydrate-binding characteristics of the VP8* external domain of the spike viral protein (Huang et al., 2012; Sun et al., 2016; Barbé et al., 2018). Divergent epidemiological studies that failed to observe associations between RVA infection and the Secretor or Lewis phenotypes may be explained by the co-circulation of strains other than P[8]-3. Indeed, either P[6] or P[8]-4 may have infected patients analyzed in Amazonia (Olivares et al., 2021), Tunisia (Ayouni et al., 2015), or Bangladesh (Lee et al., 2018). These strains attach to saliva samples regardless of the Secretor phenotype and in addition, P[6] strains preferentially infect Lewis null individuals and attach to Lewis negative saliva (Nordgren et al., 2014; Khachou et al., 2020). Therefore, failure to distinguish such strains from the analyses prevents the observation of an effect of HBGA polymorphism on the susceptibility to RV.

In agreement with observations from Pérez-Ortín et al. (2019) in Spanish hospitals, we observed that blood group O was strongly associated with a reduced risk of disease at the Nantes hospital in Metropolitan France. This was not observed in French Guiana where frequencies of ABO types were similar between the cases and controls groups. The differences between the two sites of study is likely explained by recruitment of less severe cases at Cayenne in comparison with Nantes. This was indicated by a lower clinical score at Cayenne than in Nantes as well as by a lower viral excretion. The reason why severity appears lower in Cayenne than in Nantes is unknown. A higher pathogens circulation in this tropical area may induce some degree of immunity either innate or RVA-specific, which would allow children to better control the observed infections. Alternatively, access to medical care in the community might be more restricted in Cayenne than in Nantes, leading consulting at the hospital for children presenting with less severe symptoms than in Nantes where parents first seek medical support through general practicionners or pediatricians in town. Whatever the reason for the observed lower severity in Cayenne, it strongly suggests that blood group O children are susceptible to infection by P[8]-3 strains but benefit from a decreased risk of developing severe gastroenteritis in comparison with children of non O blood groups. Indeed in Nantes, many blood group O children would be unseen since they could be taken care of by medical doctors outside the hospital emergency department. Regardless of the mechanism responsible for that effect of the ABO phenotype, considering that P[8]-3 strains are overly dominant, we showed here that in a population with a Western European distribution of the *ABO*, *FUT2* and *FUT3* alleles, in absence of vaccination, over one third of children are naturally protected from gastroenteritis of sufficient severity to warrant consulting at the hospital emergency department. This amounts to about 6,000 spared hospitalizations each year in Metropolitan France for a total population of close to 4 million children under 5 years (according to the National Bureau of Statistics),^1^ highlighting the important protecting role of HBGA polymorphisms at the population level. In addition, to their “natural” resistance to infection or severe disease, children with HBGAs null phenotypes may contribute to herd protection, akin to vaccinated children.

## Data availability statement

The original contributions presented in the study are included in the article/Supplementary material, further inquiries can be directed to the corresponding author.

## Ethics statement

The studies involving human participants were reviewed and approved by French ethic committee of OUEST V. Written informed consent to participate in this study was provided by the participants’ legal guardian/next of kin.

## Author contributions

LM, and FH recorded the clinical data. LB, TA, BL-V, CP, and SM performed laboratory assays. NR-C provided reagents and discussed the data. LM, PS, JP, and M-AV analyzed the data. JP, B-MI-M, NE, and CG conceived the work and supervised the study. LM and JP wrote the manuscript. All authors contributed to the article and approved the submitted version.

## Funding

This work was supported by an ANR-DGOS grant supporting the Gastrovim project, program CE17, grant #15-0007-01.

## Conflict of interest

The authors declare that the research was conducted in the absence of any commercial or financial relationships that could be construed as a potential conflict of interest.

## Publisher’s note

All claims expressed in this article are solely those of the authors and do not necessarily represent those of their affiliated organizations, or those of the publisher, the editors and the reviewers. Any product that may be evaluated in this article, or claim that may be made by its manufacturer, is not guaranteed or endorsed by the publisher.

## Footnotes

^1^ https://www.insee.fr/fr/statistiques/1892088?sommaire=1912926
