# Supplementary material for: Extent of the protection afforded by histo-blood group polymorphism against rotavirus gastroenteritis in metropolitan France and French Guiana
Source: Front Microbiol. 2023 Mar 10;14:1141652. doi: 10.3389/fmicb.2023.1141652 (PMC10036354; doi:10.3389/fmicb.2023.1141652)
Supplement: Supplementary file 1 [file Data_Sheet_1.docx]

# SUPPLEMENTARY MATERIALS

## **Figure S1.** Biosynthesis of histo-blood group antigens in epithelial cells.

Synthesis of ABH and Lewis antigens starts from a precursor disaccharide, type 1 precursor (boxed), that represents the terminal portion of glycolipids, O- or N-glycans of glycoproteins. The enzymes FUT2 (in bold), FUT3, A and B (A enz, B enz) denoted in red, sequentially add monosaccharides to form the A, B, H and Lewis antigens denoted in blue. Polymorphisms of the FUT2, FUT3 and ABO genes generate subgroups of people with distinct phenotypes with characteristic antigens expression (Secretor/nonsecretor, Lewis positive/Lewis negative, A, B and O). Blue squares: N-acetylglucosamine; yellow squares: N-acetylgalactosamine; yellow circles: galactose, red triangles: fucose. The figure is adapted from Le Pendu et al., Current Opinion in Virology 2014,7:88–94 and Le Pendu and Ruvoën-Clouet, Human Genetics 2020,139:903–910.

## **Figure S2:** Estimation of the population genetically protected against P[8]-3 rotavirus gastroenteritis requiring consulting at an emergency department.


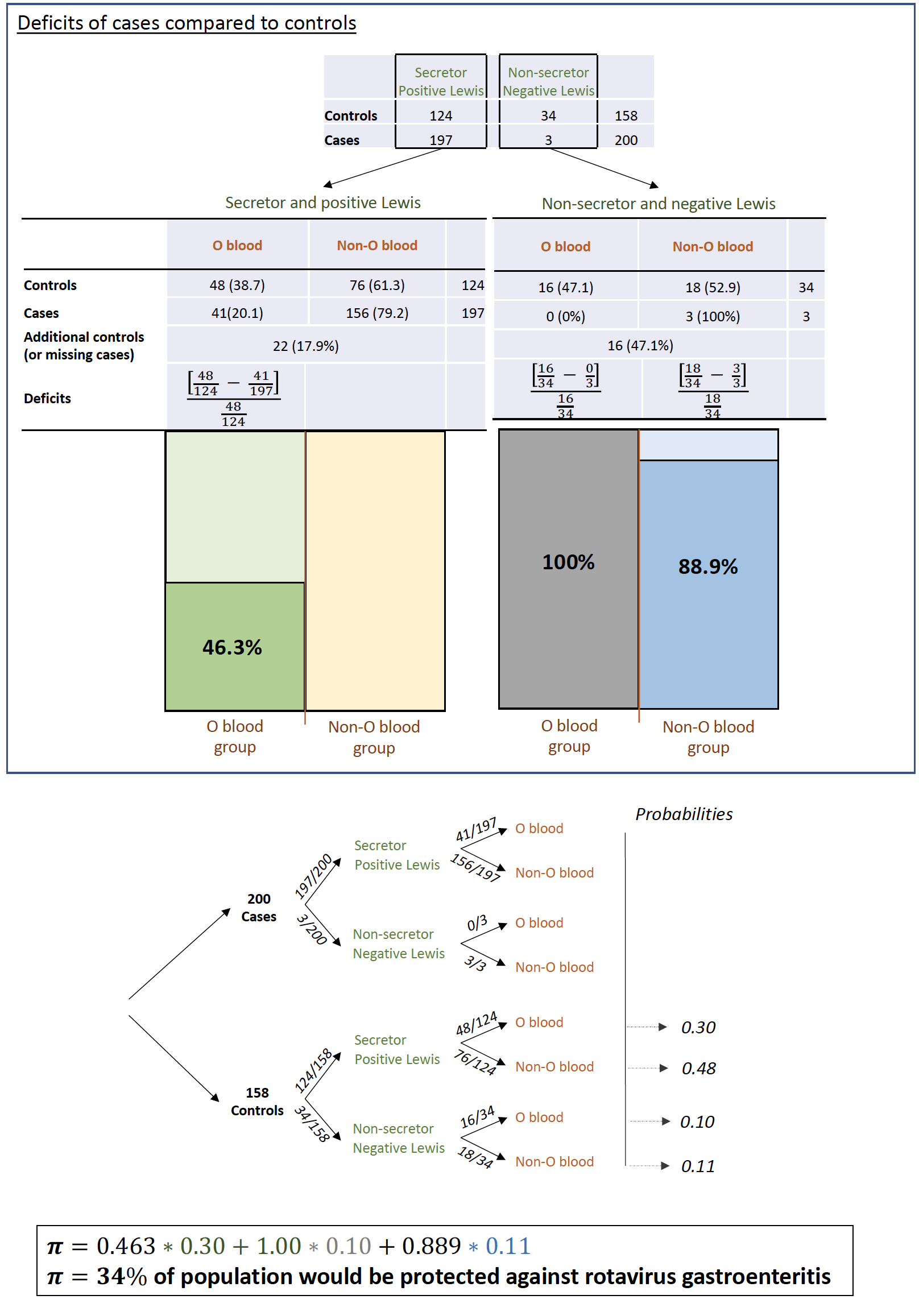


## **Figure S3:** Flow chart of participants at Nantes **(A)** and Cayenne (**B**)

**A**

**B**

| **Rotavirus primers** |  |
| --- | --- |
|  |  |
| ROTA CON3 M13F | TGTAAAACGACGGCCAGTTGGCTTCGCTCATTTATAGACA |
| ROTA CON2 M13R | CAGGAAACAGCTATGACCATTTCGGACCATTTATAACC |
|  |  |
| ROTA VP4 M13F | TGTAAAACGACGGCCAGTTATGCTCCAGTNAATTGG |
| ROTA VP4 M13R | CAGGAAACAGCTATGACCATTGCATTTCTTTCCATAATG |
|  |  |
| ROTA CON3F | TGGCTTCGCTCATTTATAGACA |
| ROTA CON2R | ATTTCGGACCATTTATAACC |
|  |  |
| ROTA VP4F | TATGCTCCAGTNAATTGG |
| ROTA VP4R | ATTGCATTTCTTTCCATAATG |
|  |  |

**References strains used for P Type Genotyping :**

Reference strains used for sub-typing the P[8]-3 clade were selected from da Silva M et al (2013) Infection Genetics and Evolution. 16: 200-205. Porcine P[19] strains were used as outgroup.

AY689211.1_Porcine_rotavirus_P19

AY689214.1_Porcine_rotavirus_P19

D38054.1_Human_rotavirus_A_P19

AY955299.1_Rotavirus_A_P6

AY955302.1_Rotavirus_A_P6

L20877.1_Rotavirus_P6

L33895.1_Human_rotavirus_A_P6

M88480.1_Human_rotavirus_P6

EF672577.1_Rotavirus_A_P4

M32559.1_Human_rotavirus_P4

M58292.1_Human_rotavirus_A_P4

DQ005111.1 Rotavirus A strain RVA/Human-wt/COD/DRC88/2003/G8P8 segment 4 P8-3.1

DQ146652.1 Rotavirus A strain RVA/Human-wt/BGD/Dhaka25/2002/G12P8 VP4 gene P8-3.1

DQ857916.1 Rotavirus A strain rj7335/03 VP4 (VP4) gene P8-3.3

DQ857917.1 Rotavirus A strain rj7363/03 VP4 (VP4) gene P8-3.4

DQ857921.1 Rotavirus A strain rj8732/04 VP4 (VP4) gene P8-3.1

EF672619.1 Rotavirus A strain RVA/Human-tc/USA/WI61/1983/G9P1A8 outer capsid protein VP4 gene P8-2

FJ947796.1 Rotavirus A human/Bethesda/DC5751/1991/G3P8 segment 4 VP4 (VP4) gene P8-1

FN179474.1 Human rotavirus A partial VP4 gene genomic RNA strain rj12077/06 P8-3.1

FN179480.1 Human rotavirus A partial VP4 gene genomic RNA strain ac11822/06/P8-3.2

GQ117014.1 Human rotavirus A isolate Nov04-H578 outer capsid protein VP4 (VP4) mRNA P8-3.5

GQ421824.1 Human rotavirus A strain sp30845/86 VP4 (VP4) gene P8-2

GQ421845.1 Human rotavirus A strain rj10998/05 VP4 (VP4) gene P8-1

HQ738605.1 Human rotavirus A isolate Omsk08-388 outer capsid protein VP4 (VP4) gene P8-3.6

JF490365.1 Rotavirus A human/Victoria/CK00029/2006/G1P8 segment 4 outer capsid protein VP4 (VP4) gene P8-3.3

JN377715.1 Rotavirus A strain RVA/Human-wt/AUS/AS07-ob V121/2007/G9P8-3.1

JX437057.1 Human rotavirus A strain 19795 VP4 gene P8-3.3

KJ454538.1 Human rotavirus A strain RVA/Human-wt/BRA/RS16838/2009/G3P8 VP8 gene P8-3.3

KM027055.1 Human rotavirus A strain sp30843-86 VP4 gene P8-2

KM027070.1 Human rotavirus A strain rj5324-02 VP4 gene P8-1

KP902533.1 Rotavirus A strain RVA/Human-wt/MWI/OP530/1999/G4P8 VP4 gene P8-4

KP902534.1 Rotavirus A strain RVA/Human-wt/MWI/OP354/1998/G4P8 VP4 gene P8-4

KP902535.1 Rotavirus A strain RVA/Human-wt/MWI/MW670/1999/G4P8 VP4 gene P8-4

**Table S1.** Crude multinomial logistic regression analyses: impact on severity of ABO blood group between cases and controls in Nantes

|  | **Severe  cases (n=155)** | **Non-severe  cases**  **(n=45)** | **Controls (n=158)** | **Crude odds ratio  [95% CI]**  *(ref=controls)* | | | |
| --- | --- | --- | --- | --- | --- | --- | --- |
|  |  |  |  | **Severe cases** | **p-value*** | **Non-severe** | **p-value*** |
| **ABO phenotype (n=358)** |  |  |  |  |  |  |  |
| Non-O blood group | 123 (79.4%) | 36 (80.0) | 94 (59.5%) | ref |  | ref |  |
| O blood group | 32 (20.6%) | 9 (20.0) | 64 (40.5%) | 0.38 (0.22 – 0.65) | **<0.01** | 0.37 (0.14 –0.84) | **0.01** |

* Fisher’s exact test

**Table S2.** Crude multinomial logistic regression analyses: impact on severity of ABO blood group between cases and controls in Cayenne

|  | **Severe  cases (n=27)** | **Non-severe cases**  **(n=21)** | **Controls (n=111)** | **Crude odds ratio  [95% CI]**  *(ref=controls)* | | | |
| --- | --- | --- | --- | --- | --- | --- | --- |
|  |  |  |  | **Severe cases** | **p-value*** | **Non-severe** | **p-value*** |
| **ABO phenotype (n=159)** |  |  |  |  |  |  |  |
| Non-O blood group | 15 (55.6%) | 10 (47.6) | 63 (56.8%) | ref |  | ref |  |
| O blood group | 12 (44.4%) | 11 (52.4) | 48 (43.2%) | 1.44 (0.56 – 3.70) | 0.48 | 1.05 (0.45 –3.47) | 1.00 |

* Fisher’s exact test
